# Cancer and the metastatic substrate

**DOI:** 10.3332/ecancer.2016.701

**Published:** 2016-12-08

**Authors:** Francisco Arvelo, Felipe Sojo, Carlos Cotte

**Affiliations:** 1 Life Sciences Centre, Institute for Advanced Studies Foundation [Fundación Instituto de Estudios Avanzado]-IDEA, Apartado 17606, Caracas 1015-A, Venezuela; 2 Tumour Biology Culture and Tissue Laboratory, Experimental Biology Institute, Central University of Venezuela, Apartado Apartado 47114, Caracas 1041-A, Venezuela

**Keywords:** cancer, metastasis, tumour micro-environment, tumour progression, tropism, exosomes, micrometastasis

## Abstract

Seventy percent of cancer patients have detectable metastases when they receive a diagnosis and 90% of cancer deaths result from metastases. These two facts emphasise the urgency for research to study the mechanisms and processes that enable metastasis. We need to develop a greater understanding of the cellular and molecular mechanisms that cause metastasis and also we need to do more. We must also consider the micro- and macro-environmental factors that influence this disease. Studying this environmental context has led us to update the ‘*seed and soil*’ hypothesis which dates back to the 19th century. This theory describes cancerous *cells* as *seeds* and the *substrate* as the *soil* in target organs though this may seem antiquated. Nonetheless, the tissue specificity that researchers have recently observed in metastatic colonisation supports the validity of the *seed and soil* theory. We now know that the metastatic potential of a tumour cell depends on multiple, reciprocal interactions between the primary tumour and distant sites. These interactions determine tumour progression. Studies of metastasis have allowed us to develop treatments that focus on therapeutic effectiveness. These new treatments account for the frequent metastasis of some tumours to target organs such as bones, lungs, brain, and liver. The purpose of this review is first to describe interactions between the cellular and molecular entities and the target organ tumour environment that enables metastasis. A second aim is to describe the complex mechanisms that mediate these interactions.

## Introduction

The genetic paradigm of cancer posits that tumours result from multiple mutations in a single normal cell. These mutations alter the genotype of the cell and transform it into a malignant phenotype [1]. The time required for this process to develop varies greatly, but it begins with the cloning of cells. Over months, years, or decades, this cloning results in the formation of a primary tumour [2]. We now recognise that malignant mutation, development, and transformation only occurs in progenitor cells called *stem cells* [3]. Mutations though in somatic cells do not produce cancer as their short half-life illustrates. Cells differentiate, mature, carry out their functions, and complete their life cycles when they die in apoptosis. The aggressiveness and metastatic power of a tumour depends on the maturity level of the stem cell that produced the mutation. Tumours derived from stem cells in early maturity will have a more heterogeneous phenotype and will metastasise quickly. Tumours derived from a more mature stem cell will have a more homogeneous phenotype and are less metastatic [4]. The biological heterogeneity of cellular populations that comprise malignant neoplasms varies widely. The notable properties of these cellular populations include their cellular surface, antigenicity, immunogenicity, proliferative index, and their sensitivity to antitumour agents. Also significant is phenotypic expression, which in combination with the aforementioned factors, allows tumours to invade other tissues.

The metastatic cascade begins in the primary tumour via local invasion characterised by several factors including the mechanical pressure exercised by proliferating tumour tissue. The action of proteolytic enzymes reduces the molecular organisation of barriers and lowers resistance to invasion. The capacity of metastatic cells to displace other cells is also a factor in the metastatic cascade [5]. This dynamic invasion process produces a Darwinian evolutionary selection in which cells acquire changes to their genetic material. These changes confer an advantage which over time becomes more common in the tumour through selection. Genetic instability thus characterises these cells and allows them to develop the capacity to invade and metastasise [6]. Metastasis develop and evolve as tumour cells spread and establish themselves in distant organs. Metastasis not only determine the prognosis and life expectancy of patients, but they also dictate the clinical outcomes of most tumours [7, 8]. Current research that examines cellular and molecular processes is critical, but we must also study the cellular, tissue, and organ environments. All of these research areas are essential to understanding cancer and finding better and more effective treatments.

## I Metastatic dissemination

*The spread of tumour cells:* Malignant tumours spread through the circulatory and lymphatic systems via the intravasation of tumour cells. Angiogenesis facilitates this process, and it results from the development of microcirculation in neo-formed vessels that form a fenestrated endothelium. These vessels are unstable intercellular unions, and form a discontinuous basal membrane that is sometimes absent [9]. Tumour progression requires that invasion coincide with an increase in vascularisation that provides nutrients and factors essential to the growth of tumour cells. Neovascularisation occurs early in tumour progression and is detectable when diseases such as ‘*in situ*’ carcinomas are incipient [10]. Tumour cells stimulate endothelial cell proliferation and vessel formation through various factors. Among the best known of these factors are *vascular endothelial growth factor,* or VEGF, IL-8, and TNF-α. Endothelial cells also produce other growth factors, such as FGF, which also promotes the growth of tumour cells [11].

Once tumour cells penetrate vessels, they can reach distant organs and proliferate. Metastasis is a highly inefficient process; even when millions of cells detach and migrate from a tumour, only a small fraction will survive and form a new tumour. This paradox is common in nature as all species use a large number of organs or specialised parts to survive. In cancer, great numbers of cells migrate from the primary tumour ensuring that some have a chance to survive and form new tumours. Although inefficient, cell migration from primary tumours is so effective that metastasis is the main cause of cancer deaths. Tumour cells that migrate may die from one of four fundamental causes. Firstly, cells are usually linked with other cells or with the surrounding environment while migrating cells are not. Consequently, migrating cells may die in a type of apoptosis that affects detached cells, *anoikis*. Tumour cells may also die because they are larger than blood cells or they may undergo cytoskeletal changes that cause cell death. Finally, migrating tumour cells are also vulnerable to immunological mechanisms [12]. The importance of epigenetic mechanisms in tumour progression is also becoming more evident, especially in the context of the cellular and extracellular environments [9]. Angiogenesis is one of the distinct and crucial events in tumour progression and cancer malignancy [13].

*Adhesiveness between normal and tumour cells:* Research has examined specifically how tumour cells adhere to the microvascular endothelium of the target organ. Specific molecules on the surfaces of both types of cells are responsible for adhesion and determine the specific site of metastasis. Three studies identified specific characteristics of circulating tumour cells, endothelia, target organs, and the microenvironment of each organ. Data from these studies indicate that these characteristics play a role in determining the sites of secondary tumour foci [14, 15, 16]. The cells capable of forming metastasis are extravasated tumour cells in close contact with blood vessels. Soluble factors secreted by tumours induce the formation of tumour foci via endothelial activation. This process is mediated by FAK (which upregulates E-selectin) and promotes the adhesion of tumour cells to the endothelium [17]. Moreover, endothelial activation by IL-1α, IL-1β, or TNF-α induces expression of E-selectin and P-selectin, as well as VCAM-1 and ICAM-1 on the surface of endothelial cells. The binding of these molecules to their ligands on tumour cells can promote contact and adhesion of tumour cells to endothelial cells [18]. Malignant cells targeting the lungs express high levels of Angpt14 and VEGF-A factors, which hinder cell-endothelial cell unions and facilitate extravasation [19]. EREG, COX2, MMP1, and MMP2 molecules also promote extravasation and metastasis [20]. Extravasation can also result from the interaction of tumour cells and platelets, in which TGF-ß activates TGF/Smad and NF-kB in cancerous cells. This process induces the epithelial-mesenchymal transition in tumour cells, stimulating extravasation [21]. Monocytes/macrophages recruited by tumour cells promote the establishment of metastatic breast cancer tumour cells in the lungs [22].

The F4/80 +, CD11b +, and Gr1 macrophages are associated with metastasis and secrete VEGF-A. This process promotes extravasation and the establishment and growth of tumour cells, possibly by increasing endothelial permeability [23]. However, soluble factors secreted by primary tumours induce recruitment of bone marrow-derived cells (BMDCs) which include immature myeloid cells, neutrophils, and monocytes. This process occurs in distant organs and results in the formation of a *premetastatic niche*, which ensures the survival and growth of tumour cells [24, 25, 26].

*Non-random metastatic distribution patterns:* Researchers have proposed two theories to explain the metastatic selectivity of tumour cells:

- *The mechanistic theory:* The relative haemodynamic position of each organ in relation to the primary tumour site determines metastatic selectivity. This theory posits that tumour cells leave the primary tumour by following the circulatory and/or lymphatic drainage routes. Tumour cells then stop at the first organ they encounter, in a process that is non-specific. Consequently, the first organ along the drainage route will be the site of the greatest number of metastases. Anatomical and mechanical factors support this theory and are significant in determining the metastatic patterns of multiple types of tumours. For example, gastrointestinal tumours start as a localised tumour in the digestive tract mucosa and grow. These tumours then invade the deepest layers of the digestive tract mucosa until reaching the serosa. When the tumour crosses the intestinal wall, it can then invade any organ within or outside the abdomen.
- *The lymphatic dissemination theory:* Lymphatic dissemination occurs when tumour cells reach the network of lymphatic vessels that surround the colon, allowing lymph drainage to multiple lymph node regions. Dissemination via the lymphatic vessel network occurs in a sequence affecting the closest lymph nodes first before spreading to more distant ones.
- *Haematogenous dissemination* occurs via tumour cells which pass into the bloodstream. Generally the first area they spread to is the liver followed by the lungs and bones because of the fact that the venous return of the intestinal tract is produced though the* portal system*. However, tumours that originate in the distal part of the rectum can give rise to an initial metastasis in the lungs because the inferior rectal vein drains into the inferior vena cava and not into the portal venous system [27].

*Organ tropism.*- In 1889, Stephen Paget [28] postulated his hypothesis classically known as ‘*seed and soil*’, asserting that the capacity of metastatic colonisation is determined by the inherent properties of tumour cells, comparing *cells* to ‘*seeds*’ and the place where they grow as the ‘*soil*’ which is the *tissue substrate*. Paget examined 735 women who had died from breast cancer and concluded that the distribution of metastases is not because of the chance that the cells were not distributed randomly, but rather because of special select microenvironment, *the tissue substrate*. He concluded that the environment played an important role in the formation and growth of the metastasis. This was backed up by experiments with animal models by injecting them with tumour cells with an affinity for a certain organ, hence, proving that the colonisation was organ specific [29, 30]. This tropism was proven experimentally with the pioneering work of Fidler and Nicolson (1976) who used two cellular variants of the murine melanoma B16: one with low growth potential, B16-F1; and the other with high growth potential, B16-F10. These cells were injected in the spleens and hearts of mice, proving that the B16-F10 cells exclusively form metastasis in the lungs, while the B16-F1 cells formed extrapulmonary metastases. These results determined that the cells did not accumulate in the first organ they found on their journey but rather the cells specifically accumulated in the lungs [31].

*Premetastatic niche preparation*.- Paget’s hypothesis gave rise to the idea that before the metastatic dissemination occurs, the cells of the primary tumour liberate cell mediators, nowadays called *exosomes*. These can travel in the circulatory system and encourage the formation of the premetastatic niche for tumour growth [32, 33]. Unlike soluble factors secreted by the cells, the exosomes contain groups of functional molecules that serve as intercellular communicators not only at a local level but also on a systemic one, apart from serving as protection for the transported molecules. The majority of the prokaryotic and eukaryotic cells liberate exosomes, including colorectal [34], lung, breast, glioblastoma, ovary, and melanoma cancer cells [35]. The exosomes are membrane vesicles that originate within the cells in *endosomal* compartments called *multivesicular bodies* [36]. Likewise, the exosomes transfer bioactive molecules to both distant and proximal receptor cells thereby altering cell’s signalling and the phenotype. Fibroblast growth factor (FGF), hepatocyte growth factor (HGF), vascular endothelial growth factor (VEGF) [37, 38], and epidermal growth factor (EGF) [39] are what stand out most along with the mutated EGFR receptors [40, 41] and HGFR [42]. It has been proven that the exosomes deriving from both normal cells and cancer cells are mediators of the metastatic process as they can promote angiogenesis [43, 44], the invasion [45, 46], and proliferation of receptor cells to support the growth of the tumour [47, 48]. It has also been suggested that exosomes that derive from cancer because of their pleiotropic character could be involved in the development and progression of the tumour through the processes that enable the following: (a) tumour cells escaping from the immune system aid in launching the inflammatory response; (b) act on the differentiation of the fibroblasts and the mesenchymal cells; (c) improve the metastatic evolution of the tumour via the promotion of the epithelial/mesenchymal transition of the tumour cells and prepare the niches of the tumour cells for their new anatomical locations [49, 50].

The exosomes of a highly metastatic melanoma increased the metastatic capacity of the primary tumour because of the influence of the progenitor cells of bone marrow via the MET receptor tyrosine kinase. These exosomes induced a vascular phenotype in the premetastatic site and reprogrammed the progenitor cells of the bone marrow that expressed c-kit, the receptor tyrosine kinase Tie2, and MET. The reduction of the expression of MET in the exosomes reduced the pro-metastatic capacity of the progenitor cells of the bone marrow. On the other hand, the progenitor cells of the bone marrow of people with metastatic melanoma with phenotype CD45 (-) C-KIT (Low/+) TIE2 (+) presented an elevated expression of MET. Likewise, the regulators of membrane transport RAB1A, RAB5B, RAB7, RAB27A (RAS-related GTP-binding proteins) and the formation of exosomes were highly expressed by the melanoma cells [41]. The integrins contained in the exosomes that participate in the formation of the premetastatic niche could be used to predict organ-specific metastasis. Exosomes of the lungs, livers, and brains of mice and humans fused with the resident cells such as the epithelial cells and lung fibroblasts, Kupffer cells and endothelial brain cells. The exosomes with integrins α6β4 and α6β1 were associated with lung metastasis, while the integrin αvβ5 was associated with liver metastasis. Likewise, it was proven that the recruitment of integrins by resident cells activates the phosphorylation of Src and the expression of the proinflammatory gene S100 (51). The human renal cancer stem cells with a phenotype marker CD105 gave rise to microvesicules that provided an angiogenic phenotype activated by the endothelial cells and promoted the formation of the premetastatic niche. These renal carcinoma cells injected into mice with severe combined immunodeficiency gave rise to the formation of lung metastasis [52]. A model of a pancreatic adenocarcinoma BSp73ASML in a rat was used to analyse the role of the CD44v variant isoform in the formation of a premetastatic niche. The CD44v is a requisite for the formation of a water-soluble matrix which in collaboration with the exosomes promotes the accumulation of leukocytes, the activation of endothelial and stroma cells, with the exosomes being the principal elements in the preparation of the premetastatic niche. The soluble fraction of the tumour serves as an exosome carrier which at the same time are carriers of chymosins and proteases necessary for the preparation of the premetastatic niche. The CD44v variant isoform could be used as a marker in tumour progression [53]. The pancreatic ductal adenocarcinoma is highly metastatic and of poor prognosis, and its exosomes induced the formation of the premetastatic niche in the liver with the subsequent formation of metastases in mice. The recruitment of exosomes derived from this tumour by the Kuffer cells induced the secretion of the transforming growth factor β and an overregulation of the production of fibronectin by the Kuffer liver cells. Initially, this gave rise to a fibrotic microenvironment and an increase in the recruitment of macrophages from the bone marrow. The macrophage migration inhibitory factor (MIF) was highly expressed by the exosomes of the pancreatic tumour and its blockage prevents the premetastatic niche formation and subsequently metastasis. In patients whose pancreatic tumours did not progress, MIF was greater in the exosomes of the patients with early stage pancreatic tumours that developed metastasis in the liver. MIF can be used as a prognostic marker in the development of liver metastasis in patients with pancreatic cancer [54].

## II Metastasis in target organs

Those organs which are frequent targets for metastases will be reviewed below. We will also give consideration to each of their particular and specific treatments. This will reaffirm the complexity of a illness that it is not lineal but contextual.

*Metastasis in the bone*.- The bones are the parts of the organism that are most affected by cancer where metastases occur from malignant breast, prostate, lung, melanoma, myeloma, kidney, and thyroid tumour types. Table 1 shows the incidence of bone metastases as a function of the primary tumor.

The predisposition of some cancers to produce metastasis in the bone, specifically in the medullary compartment, is because of the capillary structure of the bone marrow. There is slow blood flow facilitating the cells to be retained in the vascular sinusoids which are very spacious [55]. Bone marrow is a great source of growth factors such as TGF-β, IGF, of fibroblasts FGF, those derived from platelets (PDGF), morphogenic bone protein (BMPs), plus calcium which provides an appropriate environment for the growth of tumour cells [56]. A new model has been proposed to explain the preference of tumour cells for producing metastases in certain organs, affirming that not only is the bone microenvironment important, but also that the actual primary tumour produces exosomes and certain factors that prepare the target organ with a premetastatic niche in order to be able to host the disseminated tumour cells [57]. As a response to the soluble factors liberated by the haematopoietic precursor cells and the macrophages, present in the bone microenvironment, an adequate environment is created i.e. a ‘*fertile soil*’ favourable for invasion by metastatic cells [58]. The tumour cells that produce bone metastasis express the chemokine receptor CXCR4, motif chemokine receptor 4 CXC in its membrane which responds to the chemoattractant signals generated by its ligand SDF-1, also known as CXCL12. This factor is secreted both in the bone microenvironment by the osteoblasts, fibroblasts, haematopoietic stem cells, endothelial cells in the bone marrow, similar to the higher levels in the premetastatic niche. In turn the ligand SDF-1 also attracts the haematopoietic progenitor cells of the bone marrow and the endothelial progenitor cells to the metastatic site [59]. In addition, the integrin expression αvβ3 in the metastatic cells of prostate carcinoma facilitates the union to bone matrix proteins–collagen, fibronectin, vitronectin and osteopontin or OPN-, permitting the tumour cells to grow inside the bone. Not only are many factors of the bone microenvironment involved in this, but also factors secreted by the actual tumour cells [60].

With respect to the primary tumour, we see different factors are produced that stimulate the activity of osteoblasts or osteoclasts thereby giving rise to the formation of *osteolytic and osteoblastic* bone metastasis. Mixed metastases can also develop from both components. For different types of cancer, the lesions seen can be very different [61]. The metastatic cells of osteolytic types are characterised for production of an increased activity of the osteoclasts in the host giving rise to the destruction of the bone. Histologically, this is characterised by the presence of the osteoclasts eroding the bone in the tumour-bone interface [62]. Breast, lung, and kidney cancer preferably trigger the activation of the osteoclasts resulting in the appearance of osteolytic lesions [63]. The protein related to the *parathyroid hormone* (PTHrP), liberated by the metastatic cells, is the principal mediator of osteoclast activation in osteolytic metastasis. This protein stimulates the osteoblasts so that they express the ligand for the receptor activator of nuclear factor -kB or RANK more and the *osteoprotegerin* OPG less. The result is the activation of osteoclastogenesis and the increase in bone resorption. The destruction of the bone provokes the liberation of growth factors and the increase in the concentration of calcium. All these factors unite to their receptors in the membrane of the tumour cells stimulating the growth and the synthesis of PTHrP, such as multiple cytokines -like IL-1, IL-6, IL-11, IL-8-, which induce osteolysis to which the vascular endothelial growth factor or VEGF is added. This creates a viscous circle that induces the growth of tumour cells and the activity of the osteoclasts [64, 61, 62]. Bone metastasis from breast cancer affects approximately 85% of the patients with this illness. It has been found that hypoxia is associated with bone metastasis in patients with oestrogen-negative breast cancer. A high expression of lysyl oxidase LOX is associated with bone metastasis from an osteolytic lesion. LOX has been identified as a regulator of the osteoclastogenesis independent of RANK that leads to the formation of premetastatic focal lesions, which provide the platform for the tumour cells to colonise and establish as metastasis [65].

In the case of osteoblastic metastasis, this is a hallmark of prostate cancer, whose malignant cells are characterised by producing an increase in osteoblastic activity which further leads to an increase in ectopic bone formation. In this kind of metastasis the presence of a large number of osteoblasts near the tumour cells can be observed [66]. The main modulator of these metastases is the protein Endotelia-1 or Et-1, which is liberated by the metastatic cells and stimulates the proliferation of the osteoblasts through an inhibitor of the Wnt cellular signalling pathway in charge of the differentiation and activation of the osteoblasts. This inhibition is called dickkopfs or DKK1 [67]. The tumour cells also produce other osteoclast-activating factors such as BMPs and PDGF. Once they are differentiated, the osteoblasts start forming a new non-mineralised matrix on the already existing bone which contains growth factors and other proteins that attract tumour cells and enable them to survive and proliferate. The TGF-β1 is another factor that participates in the progression and development of prostate carcinoma metastasis, facilitating osteoblastic bone metastasis, and which has been proven experimentally [68]. The anti-tumour efficiency of a specific inhibitor of the kinase receptor-1 of TGF-β1, which is capable of controlling the growth of the prostate carcinoma cells in the bone [69], has also been proven. The urokinase uPA participates in the osteoblastic metastasis by having the capacity to separate and activate TGF-β which enables both the differentiation of osteoblasts and osteoclasts and also the growth of malignant cells [70]. The *prostate specific antigen* PSA also separates and activates TGF-β and PTHrP in the extreme N-terminus. This way, not only is the re-absorption mediated by PTHrP prevented, but it converts it into a stimulating factor for bone deposition [71]. Likewise, the PSA can separate IGFBP-3, the IGF-1 binding protein which cause an increase in the free IGF-1 levels, thus increasing osteoblastic activity [72].

In the treatment of prostate carcinoma, there is a close relationship with androgens which is why their neutralisation or deprivation through chemical or surgical means has become the treatment for advanced stages of the illness. The initial response to the treatment has been satisfactory as it has produced a significant clinical regression in the illness [73]. Nevertheless, despite a good response to the treatment, a relapse usually occurs after castration [74] which makes the illness progress to a phase that is insensitive to the neutralisation of hormones called *castration-resistant prostate cancer* CRPC. It determines the prognosis which can be dire with a survival rate of 16 to 18 months. The other option for these patients is chemotherapy which will be applied depending on which cells the treatment is aimed at. These are divided into three categories: 1) epithelial cells, whose treatment is based on the use of cytotoxic agents such as docetaxel and cabazitaxel [75, 76]; 2) stroma cells which include endothelial, osteoblastic, and osteoclastic cells. The cytotoxics used are thalidomide which is an anti-angiogenic [77]; bevacizuman, a monoclonal antibody against VEGF-A [78]; antrasentan, an osteoblast proliferation inhibitor [79]; denosumab, a monoclonal antibody against RANKL; the activating receptor ligand for the *nuclear factor* ϰB [80]; zoledronic acid, belongs to the group of bisphonates which inhibit the action of the osteoclasts in the prostate carcinoma cells and encourage an increase in bone density [81, 82]; 3) block the androgen receptor activation or AR which is expressed both in the prostate carcinoma cells and the microenvironment cells. For this purpose abiraterone is used which suppresses testosterone production [83], and MDV3100 or enzalutamide, an androgen receptor antagonist or AR, which blocks signalling as it inhibits its translocation to the nucleus and prevents binding to the DNA [84].

*Metastasis in the lung*.-The state of the organs has a leading role in the development of a metastatic lesion as tumour cells are more capable of developing growth points if they have a conducive microenvironment. This specification is determined by local growth factors from hormones or cytokines secreted by target organs, by adhesive interactions of the endothelium with the tumour cells, or by susceptibility in the tissue that facilitates the adhesion of the tumour cells [85]. Most likely there is a combination of the anatomical role and the specific tissue for a tendency for metastasis in the lungs [86]. The tumours that originate in the breasts, bladder, colon, kidneys, head and neck, and melanomas have a tendency to cause metastasis in the lung. According to the theory of Paget, the lung is a good *substrate* for developing metastasis because of it being a highly vascularised tissue, well oxygenated and nurtured, as well as being a tissue rich in alveolar macrophages [87]. The myeloid cells BMDC are the first defence barrier against respiratory pathogens which act against infections and secrete pro-inflammatory cytokines such as interleukin-6 and *tumour necrosis factor-alpha* or TNF-α. Both can increase permeability and tumour angiogenesis and their expression can be stimulated remotely by tumours [88]. The myeloid cells can also express VEGFR1 in a similar way to how the endothelium cells and some cancerous cells do in addition to secreting proteolytic enzymes such as MMP-9 in response to the activation of VEGFR1 through its ligands. It has been proven in knockout mice that MMP-9 and the TNF-α are crucial for metastasis in the lung as in its presence, cancerous cells can become established because of abundant pulmonary vascularisation and grow as tumour nodules [88]. Hiratsuka *et al* proposed the concept that previously preparing the lung tissue happens before metastasis actually occurs. In an experimental model to study metastases, it has been proven that the activation of the endothelium cells and lung macrophages, caused by the cells from the primary Lewis tumour or LLC (Lewis lung carcinoma), encourage the formation of metastasis in the lungs. Additionally, the suppression by VEGFR1 or MMP-9 reduces the metastasis in the lung [89]. Likewise, in the Lewis model of lung cancer, areas of the accumulation of clusters of VEGFR1, BMDC have been described along with fibronectin causing a pre-metastatic niche. These areas preferably harbour LLC cells, and it is observed that the VEGFR1 block prevented the formation of the metastasis [24, 90, 91]. Members of the VEGF family and the placental growth factor PlGF have been characterised as angiogenesis modulators in many tumours [92]. These growth factors bind to the VEGF receptors in the endothelial cells encouraging proliferation, survival, and migration. VEGF binds to VEGFR1, VEGFR2 and PNR (neuropilins), while PlGF only binds to VEGFR1, NRP-1, and NRP-2 [93]. The expression of VEGFR1 can be constitutive or induced by the expression of VEGF and PIGF caused by hypoxia which is accompanied by tumour growth [94]. On the other hand, the lysyl-oxidase, induced by hypoxia to the recruitment of the BMDC to the lungs, is a phenomenon that occurs during the formation of the metastasis. This supports the idea that the formation of the metastasis in the lung depends on the respiratory tract which include the accumulation or activation of the BMDC cells in the lung tissue [95]. By using a melanoma in an experimental model an increase in metastasis could be observed because of a high expression of the vascular cell adhesion molecule VCAM-1 in the microvasculature of the lung. An increase in the frequency of pulmonary metastasis has been observed when the animals have been inoculated with tumour cells that were previously treated with pro-inflammatory cytokines [96].

The treatment of patients with pulmonary metastasis is based on three modalities: a) prophylactic radiotherapy used in low doses on tumours in the lungs, as well as in pre-surgical pulmonary lesions; b) the resection of pulmonary metastasis through surgery which is only done when there are no extra-pulmonary metastases. It is an appropriate treatment for primary malignancy and if there is tolerance on the patient’s part, a complete recession of all the pulmonary metastases is carried out; c) chemotherapy is the standard treatment for multiple pulmonary metastases, especially in tumours with a high rate of proliferation [97]. In a murine model of breast tumour, the combined use of cyclophosphamide or CP and dabigatran etexilate, a direct inhibitor of thrombin, showed a significant synergistic effect. The tumours were significantly smaller and produced less pulmonary metastases when compared to mice only treated with one of the drugs [98]. Patients with triple-negative breast cancer for oesterogen receptor (ER)-, progesterone receptor (PR),- and HER2- have a poor prognosis as they frequently develop metastasis. The cells from this cancer, treated with selumetinib, a MEK inhibitor, showed a growth inhibition, and it was determined that significantly fewer pulmonary metastases formed in the mice than in the control mice. This MEK inhibitor therefore represents a potential alternative for the prevention of metastasis [99]. *Eribulina*, a microtubule polymerisation inhibitor, was used to measure the metastatic activity of the cells of a breast cancer ER-, PR, HER2- in an experimental model ‘*in vivo*’ with pulmonary metastasis. The pre-treatment ‘*in vitro*’ with this inhibitor caused an inversion of EMT and an induction of MET with the following reduction in the number of pulmonary metastases [100]. The ‘micro-ARN’ are conserved ARN that regulate different cellular processes whose dysfunction is involved in the development and progression of breast cancer. The chemokine receptor CXCR7 is involved in various biological processes such as cell survival, adhesions and mobility, whose over-expression has been observed in different cancers, like breast, lung, and prostate cancer. The results obtained both ‘*in vitro*’ and in a pulmonary metastasis model ‘*in vivo*’ confirmed that miR-101 is an inhibitor of growth and the invasion of the breast cancer cells through the inhibition of the CXCR7-STAT3 pathway. These results show a potential role of miR-101 as a therapeutic alternative for breast cancer [101].

*Metastasis in the brain.*- This type of metastasis is very common, making up 80% of those produced by primary lung tumours, breast cancers, and melanomas [102]. Because of the high incidence of asymptomatic brain metastasis, it is difficult to calculate the true prevalence, but different studies estimate that between 15 and 25% of cancer patients develop metastasis in the brain [103, 104]. The survival rate of patients with untreated brain metastasis is two months but could be extended to six months with conventional radiotherapy and chemotherapy treatment [105]. Numerous studies have highlighted the mechanisms that actively participate in the recruitment of tumour cells in the brain, having also established that the development of metastasis in the brain is not random. It is fundamentally a coordinated accumulation of mutations that enable the cancer cells to become established within the *central nervous system*. The carcinomas that produce metastasis in the brain have a preference for certain regions in the brain: A 80% in the cerebral hemispheres, 15% in the cerebellum, and 5% in the brain stem [106]. In the case of breast cancer, the appearance of brain metastasis usually occurs after the primary tumour has been surgically removed and after a latency period of two or three years has passed [107]. Additionally, there are different characteristics between breast cancer brain metastasis and the site of the primary tumour, expressing an increase in Ki67, a greater micro-vascular density, and the expression of micro-ARN [108, 109]. In HER-2+ breast tumours the possibility of developing brain metastasis is increased as it possibly has an affinity for neural tissue, and this occurs on average in 20% of breast cancers [110]. The difference between the primary tumour of breast carcinoma, negative for HER-2 but positive for brain metastasis, has been reported in 24% of cases which show lower survival rates [111]. The increase in the brain metastasis rate in breast cancer HER2+ highlights the tropism of the HER2-positive cells to the brain parenchyma [112, 113].

In experiments with cell lines, the overexpression of HER-2 increases the production of TGF-β, leading to the activation of TGF/Smad and the expression of E-cadherin, including snail, slug, and ZEB-1. The inhibition of HER-2 by *curcurbitacin* 2 leads to the inhibition of brain metastasis ‘*in vivo*’ [114]. On the other hand, HER-2 contributes to the epithelium/mesenchyma transition EMT through the production of TGFβ, which is its regulator [115]. The blood-brain-barrier, known by its initials ***‘bbb’***, is an obstacle for the infiltration of tumour cells because of the endothelial tight junctions. In studies using an animal model and an MRI scan, it has been shown that approximately 1.5% of the injected malignant cells formed metastasis in the brain. Among 95% of them were located throughout the brain blood vessels whilst the colonies that became established did so in the brain parenchyma. This result suggests that the basal vascular membrane represents a fertile ‘soil’ for the formation of metastasis [116, 117, 118]. The extravasation of tumour cells is facilitated by the expression of COX-2 by part of the endothelial cells which in turn induce the expression of metalloproteins by the tumour cells [119]. The overexpression of αB-crystallin by the breast cancer metastatic cells induced an increase in adhesiveness on the endothelial cells of the microvasculature of the brain [120].

The junctional adhesion molecule A or JAM-A, is a transmembrane protein that belongs to the super family of *immunoglobulins*, Ig, which is dysregulated in the breast cancer metastatic cells of the brain. Its expression is associated with the patients’ evolution and as a prognostic indicator [121]. The *alpha 2 6-sialiltranferasase* ST6GALNAC5 media, specifically in the brain metastasis and their expression in breast cancer cells, increases their adhesiveness to endothelial cells in the brain as they pass through the blood-brain barrier ***bbb***. The participation of this sialyltransferase in the brain highlights the role of the glycation in the cell surfaces of the interactions that occur in organ-specific metastasis [122]. On the other hand, the role of astrocytes in breast tumour cell survival in the brain must be highlighted. Once they have passed through the ***bbb*** they are surrounded and localised by reactive astrocytes through the overexpression of the metalloproteins or MMP, such as MMP-2 and MMP-9 [123]. In addition, the astrocytes encourage tumour growth by the secretion of cytokines, la heparanasa, neurotrophic factors, TNFα, TGFβ, IL6, etc [124]. The reactive astrocytes defend themselves against the metastatic invasion from the overexpression of the *plasmin* as its anticoagulant effect stimulates the paracrine secretion of the *cell death factor* FasL and of the inactivation of L1CAM secreted by the tumour cells. This prevents the establishment of tumour cells throughout the blood vessels. However, the tumour cells survive in the brain parenchyma protected from the plasmin by the expression in the tumour cells of the serpins, a serine protease [125]. Likewise, the astrocytes can promote the survival and growth of the metastatic tumour cells from the *neurotransmitter γ-aminobutyric* or GABA. The GABA receptors present in the breast cancer cells connect to them and then catabolise to confer a metabolic advantage for the growth of metastatic cells [126]. It is proposed that the brain parenchyma provides some kind of nourishing or growth factor that vitalises the metastatic tumour [127]. The altered metabolism is characteristic of the cancer cells. It develops a strategy which enables the metastatic brain cells to survive and proliferate. The metastatic cells do this through a process of anaerobic glycolysis, whilst normal cells do this because of the aerobic oxidation of the pyruvate [128]. Moreover, the aggressiveness of the triple negative cancer tumours (TN) are associated with a high rate of metastases to the central nervous system [129]. It has been suggested that the androgens activate the brain parenchyma astrocytes in order to facilitate the establishment of the breast cancer TN cells. This would explain why young patients with TN that present high levels of oestrogen have a higher risk of suffering brain metastasis. Possibly, the development of these metastasis could depend more on the hormonal profile of the patient than the intrinsic factors of the tumour cell [130].

The resistance to systemic therapy by metastatic brain tumours has been attributed to the ***bbb*** barrier. It was supposed that this was intact in the blood vessels of the metastatic lesion which prevented the distribution of the drugs. Nevertheless, it is now known that the presence of an intracranial metastasis modifies the vascular integrity and that the ***bbb*** then does not show normal characteristics, consequently it is known as the *blood-tumour barrier **‘btb’*** [131]. This barrier presents an increase in the permeability, reduction of blood flow, and an increased expression or reduced affluence of efflux transporters [132, 133]. The structure of the vascular network within the brain tumours is anatomically and functionally different. Anatomically the tumours promote the formation of new deformed blood vessels that lack the classical structure of the ***bbb***. The new deformed blood vessels do not have an adequate contact with the astrocytes and in addition present fenestration or pores that enable the free movement of molecules to the brain [134, 135]. In functional terms, the vascular network within the brain tumour reduces the expression of proteins of strong bonds such as ZO-1. These bonds serve as an anchor for the union of *occludin* with the endothelial membrane consequently increasing the vascular permeability [136, 137]. In adults brain metastasis clinically make up more than half of all brain tumours. The present treatment options include surgical removal, radiotherapy, and chemotherapy. Although they are curative in a low proportion, as a palliative treatment they improve survival rates and the life quality of the patients. In the case of chemotherapy, this has shown limited activity in brain metastasis in the majority of types of tumours. Above all, considering that many chemotherapeutic agents used systematically do not pass through the ***bbb*** whereas others can weaken it as they enable the extravasation of the tumour cells in circulation to the brain parenchyma [138].

Breast cancer is the second most common solid neoplasm that metastasises in the brain. Epidemiological studies show that the incidence rates of these metastases are between 10 and 16%, but autopsy reports suggest rates of up to 30% [139, 140]. The HER-2 gene is over-expressed in 20–25% of all breast cancers and makes a well-established risk for brain metastasis [141]. The use of chemotherapy in the treatment of these metastases is not only limited by the lack of drug penetration through the ***bbb*** but also by the outward flow of the drugs through the high expression of the P-glycoprotein in the endothelial cells of the brain capillaries [142]. Nevertheless, several agents have shown to have activity in this type of metastasis because of an increase in the permeability of the blood vessels deformed by the metastasis and the effects of radiation [143, 144]. The *temozolomide*, although it is permeable, has shown a limited activity in brain metastasis [145], but *cisplatin* has shown clinical activity in this metastasis associated with breast cancer as a single agent and in combination with radiotherapy [146]. *Capecitabine* is effective in breast cancer as it passes through the ***bbb*** barrier with the help of a nucleoside transporter, the hCNT [147]. Other chemotherapeutic agents used in the therapy against brain metastasis in breast cancer, that express activity anti-HER-2, are *trastuzumab* [148], *lapatinib* [149], *neratinib* [150], *afatinib* [151], *pertuzumab* [152], and *sagopilone* [153]. On the other hand, proteins that are associated to hypoxia and angiogenesis have been used as early indicators in the development of patients with metastatic breast cancer that have been previously treated with *bevacizumab*. In this treatment regimen changes in the levels of IL8 and VEGFR2 can be used to predict the response in these patients [154].

*Metastasis in the liver*.-In the liver, metastasis are the most frequent malignant processes as almost all the tumours can produce them. The most common ones are those that come from the gastrointestinal tract, especially from the colon and the pancreas, and from the breast and lung are also commonplace. In patients with colon cancer, 40% first suffer from metastasis in the liver and subsequently in the lung. The cells reach the liver through various channels, the portal system, the hepatic artery, the lymphatic vessel of the helium, or by direct extension from nearby organs [155]. Before metastases are detected certain conditions are present that make them possible: the size of the tumour cells, a fenestrated endothelium of the liver, plus the lack of a basal sub-endothelial membrane. The cancer cells extend projections through the fenestrated endothelium in the *space of Disse* establishing a direct contact with the hepatocytes [156]. The mechanisms that enable the malignant cells in the colon to form hepatic and pulmonary metastases are not well known but clinical evidence shows that different signalling pathways MAPK are involved in this process. The ERK2 activation provides the cells with the capacity to colonise the liver, whilst the reduction of the MAPK p38 signalling gives the cells the capacity to form metastasis in the lung from previously established liver lesions. The reduction in MAPK p38 signalling and the increase in the expression of parathyroid hormone like hormone (PTHLH) contribute with the extravasations of the cells in order to reach the lung. The high activity of PTHLH makes the blood vessels in the lung permeable enough to be passed by the tumour cells in order to colonise the lungs [157, 158]. It has also been shown that the inflammatory response is correlated to the metastatic potential of some tumours in the liver [155].

The liver is the third most common site of breast cancer metastasis and untreated liver metastasis shorten patient survival times to just four to eight months. In breast cancer, TNF-α can trigger e-selectin expression, increasing liver sinusoidal endothelial adhesiveness. This process is similar to the one reported for colorectal and lung cancer [159]. IL-6 induction in breast cancer cell lines reduces cellular adhesion which is associated with reduced e-cadherin expression. This finding correlates with observations of breast carcinoma patients with liver metastasis who express high levels of IL-6. Breast carcinoma cells create a pro-inflammatory microenvironment by secreting cytokines which promote adhesiveness and invasiveness in the liver [160]. Breast carcinoma cells also express chemokine receptors, such as CXCR4, while metastatic cells in the liver express CXCL12, the ligand of CXCR4. This indicates that CXCL12/CXCR4 interaction contributes to liver metastasis. CXCR4 also participates in the modulation of liver metastasis by interacting with integrins [161]. Malignant breast cells, however, also express high levels of CD44 with the highest expression occurring in cells that metastasise to the liver [162]. High levels of claudin-2 expression have also been detected in metastatic breast cells in the liver. These cells metastasise by adhering to extracellular matrix proteins, such as fibronectin and type IV collagen, which are abundant in the liver [163].

In primary colon tumours, aberrant TGFα/EGFR expression helps propagate tumour cells in lymph nodes and the liver [164]. Colon cancer cells express high amounts of EGFR and overexpress TGFα in response to hypoxia, triggering a signalling cascade that helps them survive. Cellular proliferation thus involves Ras/MAPK and anti-apoptotic activity (phosphatidylinositol-3-kinase [PI3K] / Akt) which ultimately correlates with metastasis and resistance to chemotherapy [165, 166]. ER, PR, and HER-2 status is also essential to determining the response to any kind of treatment whether adjuvant hormonal therapy, directed molecular therapy, or chemotherapy. Researchers have evaluated and established the relationship of ER, PR, and HER-2 in primary breast cancer and metastatic cancer in specific organs [167]. For example, one study found that increased HER-2 phosphorylation is a critical factor in establishing liver metastasis in breast cancer [168].

Chemotherapy and hormones remain alternatives for treating breast cancer patients with liver metastasis. Surgery is another option for palliating metastatic complications, and although it is limited to individual liver segments only. Nonetheless, partial removal of the liver offers the possibility of a cure. HER-2-positive invasive ductal carcinoma patients with liver metastasis have achieved complete response through a combination of treatments. These patients took docetaxel and two anti-HER-2 agents, trastuzumab and pertuzumab, along with surgery to resect the metastatic liver part [169]. Another option for treating patients with liver metastasis is to administer a combination of drugs via hepatic arterial infusion (HAI): irinotecan, bevacizumab, and oxaliplatin combined with bevacizumab or cetuximab [170] have been used. In colorectal cancer patients with liver and lung metastases, completely resecting metastases in both organs offers the best survival results. A retrospective study compared general three- and five-year survival rates in three groups of colorectal cancer patients with liver and lung metastases. Patients who received chemotherapy and had surgery to resect only liver metastasis had higher survival rates than patients who only received chemotherapy. The former group, however, had a lower survival rate than patients who received chemotherapy and had surgery to resect both liver and lung metastases [171, 172]. An experimental model demonstrated the involvement of *laminin receptor* LRP/LR in cancer progression. This model established the role of LRP/LR in adhesion and invasion in HUH-7 liver cancer cells. These cells showed high levels of LRP/LR compared to K562 leukaemia cells and MCF-7 breast cells. Treating liver cells with the anti-LRP/LR specific antibody IgG1-iS18 significantly reduced the adhesive potential of liver cells to laminin-1 and their invasive potential. This result suggests the use the IgG1-iS18 antibody as a therapeutic alternative in liver metastasis [173]. Another study used an animal model to examine the combined effect of SiARN targeting the hGHR growth hormone receptor and 5-fluorouracil on colon cancer metastases. The results of that study showed that this combination reduced the incidence of liver metastasis in colon cancer [174]. A study of 46,027 patients examined metastatic distribution patterns in colon and rectal cancer. This study found that the incidence of liver metastasis was 13.8% in colon cancer patients versus 12.3% in those with rectal cancer. The incidence of lung metastasis was 5.6% in rectal cancer patients and 3.7% in those with colon cancer. The incidence of bone metastasis in rectal cancer patients was 1.2% and 0.8% for colon cancer patients. This study also examined the incidence of bone metastasis in colorectal cancer patients. Incidence was 10.0% in patients with lung metastasis, but 4.5% in patients without lung metastasis. Brain metastasis rates were 3.1% and 0.1%, respectively. Understanding metastatic distribution patterns can help clinicians apply chemotherapy more effectively, and is also valuable for possible surgical treatment [175]. Another study examined the benefits of surgical resection in patients with stage IV metastatic colon cancer. Patients received several cycles of neoadjuvant chemotherapies, such as combinations of folinic acid, fluorouracil (5-FU), and oxaliplatin (FOLFOX); folinic acid, (5-FU), and irinotecan (FOLFIRI); folinic acid, (5-FU), oxaliplatin, and irinotecan (FOLFOXIRI); and bevacizumab, panitumumab, and cetuximab. Patients who were candidates for immediate surgery did not benefit from treatment, while patients who were not candidates for surgery showed a survival benefit from chemotherapy [176]. Another study evaluated the efficacy of treating colon cancer patients with liver metastasis using electromagnetic waves. This treatment was effective in increasing patient survival times and controlling liver metastasis measuring 3 cm or less [177].

Researchers now use models that aim to mimic morphological structures and the relationships that exist in living systems. This requires conducting studies using three-dimensional models that approximate the cellular and molecular composition of tumours. Researchers grow *multicellular tumour spheroids*, or MTS [178], seed porous scaffolds [179], and create miniature versions of various organs connected by vascular canals called ‘Chips’ [180]. Such models will help researchers develop therapies that act on tissues and systems and not just at the cellular and molecular levels. These advances will make research and clinical practice more efficient, optimise prevention, and create better treatments.

## Conclusions

Based on new research findings, the ‘*seed and soil*’ hypothesis has helped cancer researchers to reassess and create a new framework for analysing metastasis. We now have a more advanced perspective on this complex and multifactorial disease. We see that cancer and its malignancies do not result solely from specific genetic mutations in tumour cells. Moreover, we recognise the importance of studying the tumour macroenvironment in order to achieve a comprehensive understanding of the manifestations of cancer and how to treat it. Any study that focuses only on cells and genes will thus be incomplete as not all people with altered genes develop cancer. Current accepted treatments based on conventional protocols have poor efficacy as they focus on destroying tumours without evaluating cancer patients more comprehensively. Clinicians use the same treatments for all patients with the same histological type of tumour, not considering that tumour progression varies for all patients. We must consider that surrounding tissues consisting of non-cancerous cells including blood vessels and immune system cells play an important role in cancer. Patients require treatments that also focus on the tumour environment in order to develop preventive therapies that make that environment adverse for tumour cells. We must therefore understand the tumour environment in order to clarify our view of this disease and help us use treatments more rationally.

## Figures and Tables

**Table 1.** Incidence and prognosis of bone metastases of different types of cancer.

	Incidence Rate of Illness	Median Survival Rate (months)	Five- Year Survival Rate
Myeloma	95–100%	20	10%
Breast	65–75%	24	20%
Prostate	65–75%	40	25%
Lung	30–40%	<6	<5%
Kidney	20–25%	6	10%
Thyroids	60%	48	40%
Melanoma	15–45%	<6	<5%

Data by Rubens and Coleman
